# Experimental strategies to achieve efficient targeted knock-in via tandem paired nicking

**DOI:** 10.1038/s41598-021-01978-w

**Published:** 2021-11-19

**Authors:** Md. Lutfur Rahman, Toshinori Hyodo, Sivasundaram Karnan, Akinobu Ota, Muhammad Nazmul Hasan, Yuko Mihara, Md Wahiduzzaman, Shinobu Tsuzuki, Yoshitaka Hosokawa, Hiroyuki Konishi

**Affiliations:** 1 Department of Biochemistry, Aichi Medical University School of Medicine, 1-1 Yazako Karimata, Building #2, Room 362, Nagakute, Aichi 480-1195 Japan; 2 Present Address: Bangladesh Medical Research Council, Dhaka, 1212 Bangladesh; 3 Present Address: Eukaryotic Gene Expression and Function (EuGEF) Research Group, Chattogram, 4000 Bangladesh

**Keywords:** Genetic engineering, CRISPR-Cas9 genome editing

## Abstract

Tandem paired nicking (TPN) is a method of genome editing that enables precise and relatively efficient targeted knock-in without appreciable restraint by p53-mediated DNA damage response. TPN is initiated by introducing two site-specific nicks on the same DNA strand using Cas9 nickases in such a way that the nicks encompass the knock-in site and are located within a homologous region between a donor DNA and the genome. This nicking design results in the creation of two nicks on the donor DNA and two in the genome, leading to relatively efficient homology-directed recombination between these DNA fragments. In this study, we sought to identify the optimal design of TPN experiments that would improve the efficiency of targeted knock-in, using multiple reporter systems based on exogenous and endogenous genes. We found that efficient targeted knock-in via TPN is supported by the use of 1700–2000-bp donor DNAs, exactly 20-nt-long spacers predicted to be efficient in on-target cleavage, and tandem-paired Cas9 nickases nicking at positions close to each other. These findings will help establish a methodology for efficient and precise targeted knock-in based on TPN, which could broaden the applicability of targeted knock-in to various fields of life science.

## Introduction

Targeted knock-in is a type of genome editing technology that allows for programmed insertion, deletion, and substitution of DNA sequences within the cellular genome. Targeted knock-in using a foreign donor DNA fragment as a template is one of the best-studied, most versatile, and practical technologies for such programmed genome manipulation, and it has shown promise in various fields of life science including genetic medicine^1,2^. Recently, we and others developed and characterized a strategy for targeted knock-in mediated by Cas9 nickases, in which Cas9 nickases introduce nicks but not double-strand breaks (DSBs) into both the genome and donor DNAs^3–5^. As DSBs remain absent, this strategy allows for targeted knock-in without creating appreciable promiscuous alterations, *i.e.*, random insertions and deletions (indels), within the genome. Moreover, this strategy was proven to support targeted knock-in with an efficiency largely equivalent to, or even higher than, that achieved by the conventional method of targeted knock-in based on DSBs created by Cas9 nucleases.

One methodology aligned with the nicking-based strategy for targeted knock-in is tandem paired nicking (or tandem nicking; hereafter denoted as TPN)^3–5^, in which the target sites of two Cas9 nickases are placed on the region of homology between the donor DNA and the genome in such a way that the target sequences of two Cas9 nickases encompass the knock-in site and the nicks are introduced in the same DNA strand (Supplementary Fig. S1). Thus, a total of four nicks (two in the donor DNA and two in the genome) are created in this method, enabling precise and substantially efficient targeted knock-in. Targeted knock-in via TPN has been shown to neither significantly activate the p53 signaling pathway nor be appreciably restricted by wild-type p53 function, which is in contrast to Cas9 nuclease-based targeted knock-in^5–7^. In addition, unlike many other methodologies for targeted knock-in, TPN does not require the incorporation of additional nucleotide changes, such as silent mutations, into donor DNAs. This property of TPN allows to introduce only the intended genomic changes while precluding additional, unnecessary nucleotide changes upon targeted knock-in; hence, TPN enables users to conduct knock-in experiments exactly as desired. Therefore, TPN will be useful for editing gene promoters, enhancers, non-coding RNAs, and intergenic regions, in which additional nucleotide changes may result in unexpected consequences for the function of edited DNA regions. The use of TPN can also be advantageous for the editing of coding genes because transcription, splicing, translation, and degradation of mRNAs originating from edited genes are potentially affected by the genomic incorporation of additional nucleotide changes, even when they are silent.

As another advantage of TPN-based targeted knock-in, Cas9 nickases designed against genomic sites fairly distant from the knock-in sites (up to 416-bp distant in *CD55* gene targeting), albeit within the region of homology, are capable of promoting relatively efficient targeted knock-in^5^. Such a broad genomic window for locating Cas9 target sites permits large flexibility when designing experiments for TPN-based targeted knock-in compared with the conventional Cas9 nuclease-based approach. Paradoxically, however, this flexibility may pose a challenge when attempting to identify an optimal pair of Cas9 nickases with which to conduct TPN-based targeted knock-in with the highest achievable efficiency, because guidelines for choosing optimal Cas9 nickases for TPN are currently unavailable.

In the present study, we sought to determine a design for TPN experiments that allows researchers to conduct efficient TPN-based targeted knock-in. To achieve this goal, we evaluated several experimental factors for TPN, including the choice of protospacers among multiple candidate genomic sites, the design of single guide RNAs (sgRNAs) created against the chosen protospacers, and the amounts of plasmids expressing Cas9 (D10A) and sgRNAs that are to be transfected into cells. We also attempted to determine the length and amounts of donor DNAs required to achieve efficient TPN-based targeted knock-in.

## Results

### A 1700–2000-bp homologous region in a donor DNA is required for efficient targeted knock-in via TPN

**Figure 1:**
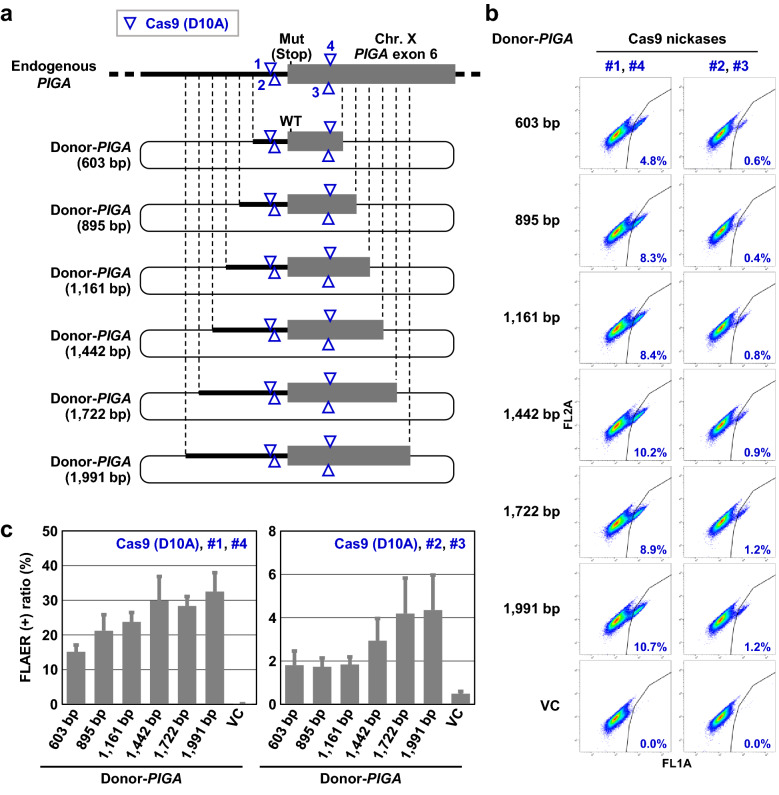
Efficient targeted knock-in via TPN is supported by 1700–2000-bp donor DNAs. **(a)** Donor plasmids carrying various lengths of homology with a genomic region surrounding the human *PIGA* intron 5/exon 6 boundary. The lengths of homology in respective donor plasmids (Donor-*PIGA*) are noted in parentheses. Numbers from 1 through 4 indicate distinct Cas9 nickases and correspond to #1 through #4 in (**b**,**c**). Hereinafter, bold lines and gray boxes in schematic representation indicate portions of *PIGA* intron 5 and exon 6, respectively, unless otherwise noted. Thin and dotted lines indicate backbones in plasmids and homologous regions, respectively. Mut (Stop): a nonsense mutation; WT: wild-type sequence. **(b)** Representative dot graphs obtained from FCM analyses. Percentages of FLAER-positive cells are denoted in the graphs. VC represents a vector control. Hereinafter, FL1A and FL2A indicate fluorescence signals measured at 530 ± 15 nm and 585 ± 21 nm, respectively, under 488-nm laser excitation. **(c)** Graphical representation of the results. Data represent mean and SEM values from three independent experiments.

Our previous study demonstrated that the length of homology between donor DNAs and the genome affects the efficiency of targeted knock-in significantly more in TPN than it does in the Cas9 nuclease-based approach^5^. In the present study, we initially attempted to clarify the appropriate length of homology required to achieve high efficiency in targeted knock-in via TPN. To this end, we generated multiple donor plasmids bearing homology of different lengths with a genomic region surrounding the human *PIGA* intron 5/exon 6 boundary (Fig. 1a). These plasmids were then transfected, together with a Cas9 nickase pair, into a cell clone derived from a human colon cancer cell line HCT116 in which a truncating mutation had been incorporated into *PIGA* exon 6 (termed the HCT116-mut*PIGA* clone). The transfection of this cell clone with the aforementioned plasmids elicits TPN-based targeted knock-in, and the successful knock-in of the wild-type *PIGA* sequence into the cellular genome results in the reactivation of mutant *PIGA*. Functional *PIGA* in cells is readily detectable when the GPI-anchors that protrude from cell membranes are stained with fluorescence-labeled inactive aerolysin (FLAER) and the cells are analyzed by flow cytometry (FCM)^8^.

As the results of this experiment, we found that the use of longer homologous regions overall improved the efficiency of targeted knock-in via TPN, consistent with our previous results (Fig. 1b,c)^5^. Meanwhile, using two different Cas9 nickase pairs, we detected no significant differences in the efficiency of targeted knock-in via TPN when comparing the use of 1722-bp and 1991-bp homologous regions. These data indicate that the optimal length of homologous region in donor plasmids to achieve high efficiency of TPN-based targeted knock-in should be approximately 1700–2000 bp.

In this experiment, nicking with sgRNAs #1 and #4 (sense strand) apparently permitted more efficient TPN-based targeted knock-in than did nicking with sgRNAs #2 and #3 (antisense-strand). To investigate the reason for this, we generated four Cas9 nucleases targeted to the same protospacers as those of the Cas9 nickases employed in this experiment, and quantified the cleaving capacity of these Cas9 nucleases by transfecting them into 293T cells and performing an indel detection by amplicon analysis (IDAA). As a result, we found that a Cas9 nuclease containing sgRNA #4 cleaves DNA strands more efficiently than that containing sgRNA #3 (Supplementary Fig. S2). These data suggest that a Cas9 nickase containing sgRNA #4 likely has greater nicking activity than one containing sgRNA #3, and that the observed difference in the efficiencies of TPN-based targeted knock-in achieved with two different Cas9 nickase pairs is at least partly attributable to the different cleaving capacities of Cas9 nickases.

### Short distances between nicks as well as high CRISPick on-target efficacy scores support efficient targeted knock-in via TPN

**Figure 2:**
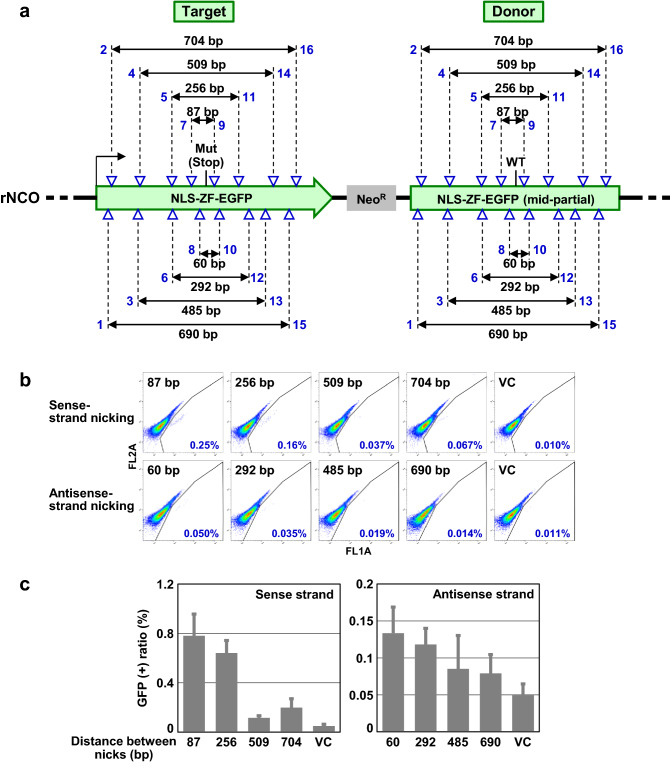
Shorter distance between two nicking sites promotes greater efficiency of targeted knock-in via TPN. **(a)** rNCO, a GFP-based reporter construct monitoring the incidence of intramolecular targeted knock-in. Targeted knock-in of the wild-type sequence within the “Donor” (right) into the mutated site within the “Target” (left) results in the reconstitution of a full-length wild-type EGFP. The numbers from 1 through 16 associated with triangles, from left to right, indicate the positions nicked by Cas9 nickases. NLS: nuclear localization signal; ZF: zinc finger; Neo^R^: neomycin resistance gene. **(b)** Representative dot graphs obtained in FCM analyses. **(c)** Graphical representation of the results. Data represent mean and SEM values from three independent experiments.

We next assessed whether the distance between two nicks placed on the region of homology between donor DNAs and the genome significantly affects the efficiency of targeted knock-in via TPN. In this analysis, we leveraged rNCO, a GFP-based reporter construct that allows monitoring of TPN-based targeted knock-in via a GFP signal (Fig. 2a). The EGFP gene was chosen as the substrate for DNA recombination in this assay because a large number of protospacer adjacent motifs (PAMs) are available and well distributed over the EGFP coding sequence. Cell clones harboring a single copy of rNCO within the genome have been established previously^5^.

We generated a total of 16 Cas9 nickases (*i.e.*, Cas9 (D10A) associated with 16 different sgRNAs): eight nicking the sense strand and eight nicking the antisense strand of the EGFP gene. The generated Cas9 nickases were transfected into an HCT116-derived cell clone carrying rNCO (HCT116-rNCO), and the incidence of TPN-based EGFP recombination was quantified by FCM. This assay demonstrated that a shorter distance between two nicks on the homologous region generally led to higher efficiency in TPN-based targeted knock-in, both in sense- and antisense-strand nicking (Fig. 2b,c).

**Figure 3:**
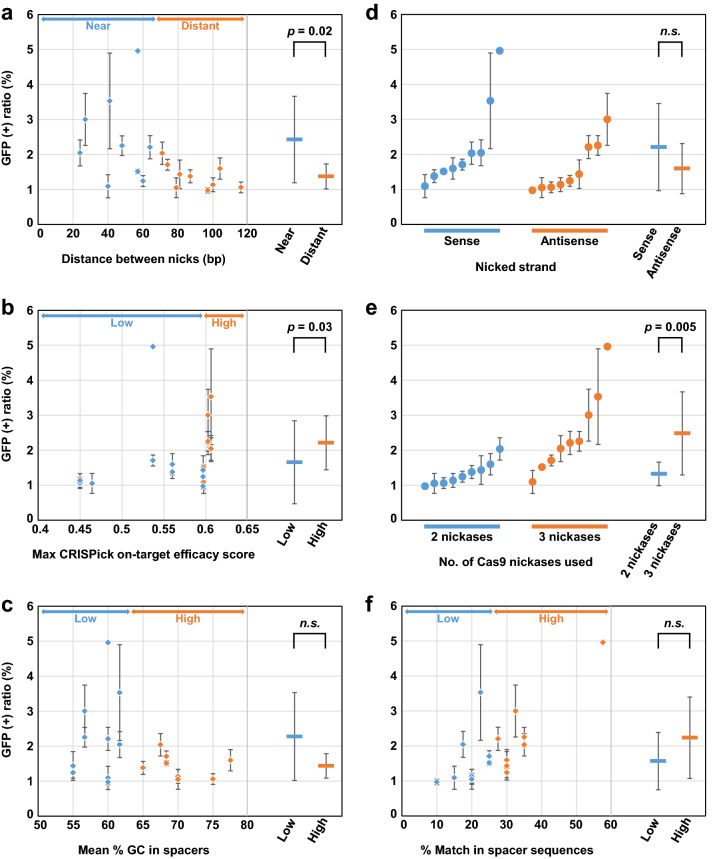
Higher CRISPick on-target efficacy scores, in addition to a shorter distance between two nicks, is a key factor to conduct efficient targeted knock-in via TPN. An HCT116-based reporter cell clone, HCT116-rNCO, was transfected with Cas9 nickases designed against the EGFP gene within a 70-bp range from the knock-in site, and analyzed by FCM (also see Supplementary Fig. S3a,b for experimental details). The ratios of GFP-positive cells are plotted on the graphs with x-axes indicating **(a)** distances between nicks, **(b)** the maximum CRISPick on-target efficacy scores among those of two or three spacers used, **(c)** the mean percentages of GC content in two or three spacers, **(d)** nicked strands, **(e)** the numbers of Cas9 nickases used, and **(f)** the percentages of identical nucleotides within spacers. In (**f**), when three Cas9 nickases were used and two spacers designed against the same position in the wild-type and mutant EGFP, respectively, showed different percent identities to the remainder of three spacers, the average percent identity was calculated and used for data plotting. In (**a**–**f**), the average data obtained from three independent experiments were determined for individual 18 sgRNA combinations [depicted by squares (**a**–**c**,**f**) or circles (**d**,**e**)], divided into two groups at the median values on x-axes (**a**–**c**,**f**) or by the indications on x-axes (**d**,**e**), and then statistically analyzed as summarized to the right in the respective graphs. All error bars in (**a**–**f**) represent standard deviations.

We then generated additional Cas9 nickases within a short distance range (~ 70 bp) from the knock-in site, and assessed the efficiency of TPN-based targeted knock-in achieved using pairs of generated Cas9 nickases (Supplementary Fig. S3a,b). When a nick was created very close to the knock-in site so that the protospacer of the Cas9 nickase overlapped with the knock-in site, two versions of the Cas9 nickase (one nicking a mutant EGFP in the “target” and the other nicking a wild-type EGFP in the “donor”) were used in combination, *i.e.*, a total of three Cas9 nickases were used to conduct TPN in such cases (Supplementary Fig. S3b). Univariate analysis of the results from this assay confirmed that the efficiency of targeted knock-in via TPN is higher when the distance between two nicks on the homologous region is shorter (Fig. 3a). In addition, targeted knock-in was more efficient when TPN was conducted using Cas9 nickases with higher sgRNA on-target efficacy scores^9^, which were determined using the CRISPick website (https://portals.broadinstitute.org/gppx/crispick/public; Fig. 3b). In contrast, the efficiency of TPN-based targeted knock-in was not significantly associated with either GC content in the spacer sequences of Cas9 nickase pairs (Fig. 3c) or nicked strands (Fig. 3d). Although the use of three Cas9 nickases led to significantly higher efficiencies in targeted knock-in via TPN than the use of two Cas9 nickases (Fig. 3e), this likely occurred because pairs of Cas9 nickases separated by very short distances require the use of three Cas9 nickases due to the overlap between a protospacer sequence and the knock-in site.

We additionally used another rNCO reporter cell clone derived from MCF10A (MCF10A-rNCO), an immortalized noncancerous human breast epithelial cell line, in the same analyses described above using 18 combinations of Cas9 nickases. The results of this assay were consistent with the findings obtained using HCT116-rNCO (Supplementary Fig. S4a–e).

While conducting the above assays using HCT116-rNCO (Fig. 3), we found a set of apparent outlying data that reached approximately 5% GFP positivity with a single combination of Cas9 nickases (S-2 and S-5 shown in Supplementary Fig. S3a). This exceptionally high efficiency of targeted knock-in was repeatedly observed in three independent experiments. In an attempt to identify the cause of these outlying data, we found that the spacer sequences of this Cas9 nickase combination had remarkable homology with each other, particularly at their 3′ regions, at which eight of 10 nucleotides (so-called “seed sequences”) were identical in each spacer (Supplementary Fig. S3c). We thus postulated that the percentage of identical nucleotides in two spacer sequences of Cas9 nickase pairs may significantly affect the efficiency of targeted knock-in via TPN, and addressed this possibility using the dataset with 18 Cas9 nickase combinations described above. However, only a marginal, non-significant association was detected between the efficiency of TPN-based targeted knock-in and the ratios of identical nucleotides in spacer sequences. In addition, this marginal relationship was detected with data from HCT116-rNCO but not from MCF10A-rNCO (Fig. 3f and Supplementary Fig. S4f.), suggesting that the marginal effect of identity between two spacer sequences on the efficiency of TPN-based targeted knock-in likely depends on the cellular context.

### Further evidence for the advantage of locating two nicks close together to achieve efficient targeted knock-in via TPN

**Figure 4:**
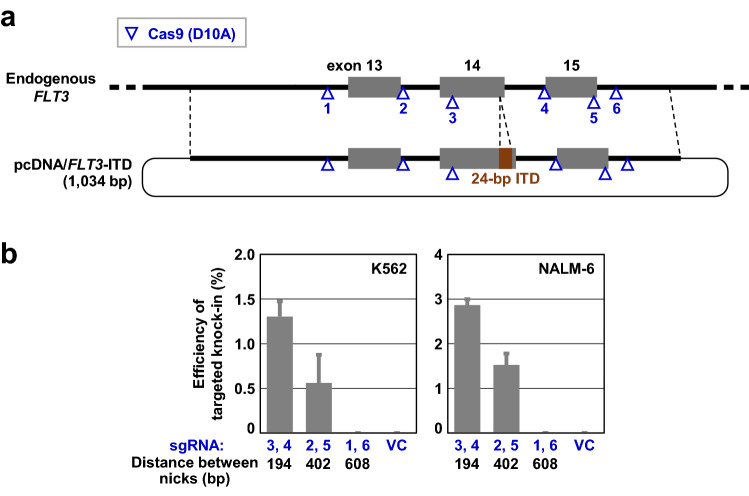
An *FLT3*-based assay providing further evidence that locating two nicks close together facilitates TPN-based targeted knock-in. **(a)** Experimental setup of targeted knock-in of an *FLT3*-ITD. The length of a homologous region in the donor plasmid (pcDNA/FLT3-ITD) is noted in parenthesis. ITD, internal tandem duplication. **(b)** The efficiencies of targeted knock-in of the 24-bp ITD in the K562 (left) and NALM-6 (right) cell lines determined by IDAA assays. IDAA, indel detection by amplicon analysis. Data represent mean and SEM values from three independent experiments.

To further confirm the negative correlation between the achievable efficiency of TPN-based targeted knock-in and the distance between two Cas9 nicking sites, we employed another assay system that allowed us to quantify the efficiency of targeted knock-in. *FLT3* gene alterations occur frequently in acute myelogenous leukemia cases and sporadically in other myeloproliferative diseases^10,11^. K562 and NALM-6 are model hematopoietic cell lines commonly used to assess pathogenic roles for the alterations and high-level expression of *FLT3*^12–17^. We thus chose to introduce an *FLT3*-internal tandem duplication (ITD), a typical pathogenic alteration in the *FLT3* gene, into the genomes of the K562 and NALM-6 cell lines via TPN-based targeted knock-in. These cell lines were transfected with pcDNA/*FLT3*-ITD^17^, a donor plasmid bearing a 1010-bp *FLT3* region with a 24-bp-long ITD (1034 bp in total), along with three Cas9 nickase pairs with various distances between nicking sites (Fig. 4a). Subsequently, the efficiencies of targeted knock-in achieved were quantified by IDAA. Although the region of homology in this assay system was shorter than the optimal length defined above (1700–2000 bp), we found, in both cell lines, that ITD incorporation into the genome occurred depending on the proximity of two Cas9 nicking sites (Fig. 4b). These data provide further evidence that a short distance between two nicking sites promotes relatively efficient targeted knock-in via TPN.

### Transfection with a large amount of donor DNA supports efficient targeted knock-in via TPN

**Figure 5:**
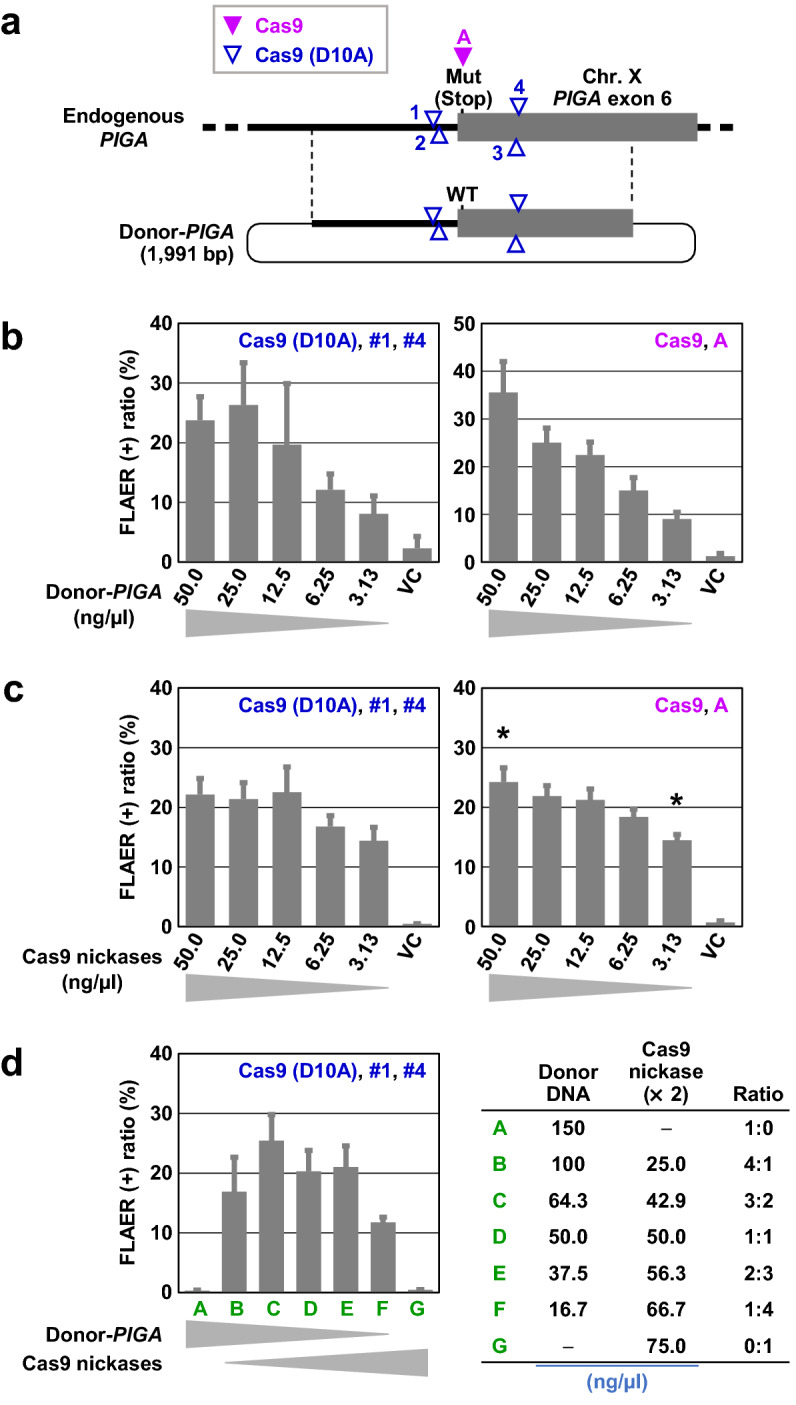
Transfection with a large amount of a donor plasmid leads to efficient targeted knock-in via TPN. **(a)** Scheme showing the experimental setup of the assay. **(b)** The efficiency of targeted knock-in achieved using different concentrations of Donor-*PIGA*. **(c)** The efficiency of targeted knock-in achieved using different concentrations of Cas9 plasmids. In (**c**), a statistically significant difference in the efficiency of targeted knock-in was observed only between the transfection of 50 ng/µL and 3.13 ng/µL plasmids expressing the Cas9 nuclease (*; *p* = 0.02) in a concentration range of 3.13–50.0 ng/µL. In (**b**,**c**), when concentrations of Donor-*PIGA* and Cas9 plasmids are not denoted on the graphs, these plasmids were used at 50 ng/µL, and the resultant plasmid solutions were supplemented with pBluescript II KS ( +) up to 150 ng/µL. **(d)** The efficiency of targeted knock-in achieved by transfecting various concentrations of Donor-*PIGA* and plasmids expressing Cas9 nickases. The sum of three plasmid concentrations was fixed at 150 ng/µL as shown to the right of the graph. Data obtained with experimental conditions B–F were not significantly different. In (**b**–**d**), 50 ng/µL plasmids (1 μg plasmids dissolved into 20 µL electroporation buffer per sample) correspond to 15.5 nM (Donor-*PIGA*; 4895 bp including a 1991 bp homologous region) or 8.3 nM (Cas9 plasmids; 9178 bp) in molar concentration. Data represent the mean and SEM values of three independent experiments.

We next sought to determine the optimal amounts of donor DNA and plasmids expressing Cas9 nickases that elicit efficient targeted knock-in via TPN when transfected into cells. We used the HCT116-mut*PIGA* clone as an experimental platform in this assay (Fig. 5a). Because this clone was transfected by electroporation in our experiments, we assessed the relationship between the concentration of plasmids dissolved in electroporation buffer and the efficiency of targeted knock-in achieved via TPN. Overall, the efficiency of targeted knock-in was positively correlated with the concentration of Donor-*PIGA*, both in TPN-based and Cas9 nuclease-based targeted knock-in (Fig. 5b). In contrast, the concentration of PX462-based constructs expressing Cas9 nickases did not significantly affect the efficiency of targeted knock-in via TPN in a concentration range of 3.13–50.0 ng/µL, and similar results were obtained for Cas9 nuclease-based targeted knock-in (Fig. 5c). The different effects of the concentrations of Donor-*PIGA* and PX462-based constructs on the efficiency of targeted knock-in may be attributable to the fact that Donor-*PIGA* serves as a recombination substrate for itself, whereas PX462-based constructs serve as transcriptional templates for the production of Cas9 (D10A) and sgRNA molecules.

In further analyses, we selected several concentrations of Donor-*PIGA* and PX462-based constructs in such a way that the sum of the plasmid concentrations was fixed at 150 ng/µL. We then transfected the plasmids into the reporter clone and performed FCM analyses of the cells (Fig. 5d). The obtained data showed that the use of a 1.5-fold concentration (*i.e.*, 2.8-fold molar concentration) of Donor-*PIGA* relative to the PX462-based constructs gave the highest mean efficiency of targeted knock-in via TPN, although the efficiencies of targeted knock-in did not significantly differ among various concentrations of plasmids (B–F in Fig. 5d). These data collectively indicate that a higher concentration of donor plasmid relative to that of Cas9 nickase-expressing plasmids should be used for efficient targeted knock-in via TPN.

### Spacers exactly 20 nt in length support efficient targeted knock-in via TPN

**Figure 6:**
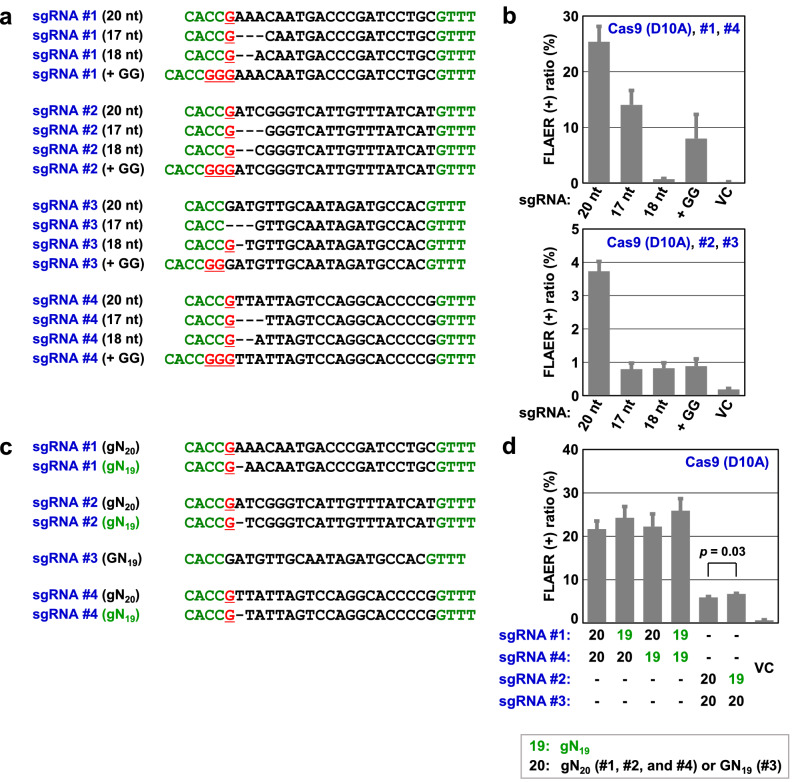
The use of spacers exactly 20 bp in length supports efficient targeted knock-in via TPN. The HCT116-mut*PIGA* clone was transfected with Donor-*PIGA* (1991 bp) along with tandem-paired Cas9 nickases loaded with various sgRNAs. **(a,c)** Lists of spacer sequences incorporated into PX462. “CACC” and “GTTT” located at the 5′ and 3′ ends of each sequence, respectively, are cohesive overhangs for ligation of DNA fragments. Underlined Gs indicate artificially integrated deoxyguanosines that are not present at the corresponding positions in the genome. **(b,d)** Graphical representation of data acquired by FCM analyses using sgRNAs that contain spacers listed in (**a,c**). Data represent the mean and SEM values of three independent experiments.

Modifications on sgRNAs, specifically the truncation of spacers from 20 nt to 17 or 18 nt, or the addition of two deoxyguanosines at the 5′ ends of spacers, were reported to reduce off-target DNA cleavage by Cas9 nuclease and thereby increase the specificity of genome editing^18–21^. It was also reported that the efficiency of targeted knock-in via the Cas9 nuclease-based approach is often improved by these modifications on sgRNAs^22,23^. Consequently, we assessed whether such modifications on sgRNAs similarly improve the efficiency of TPN-based targeted knock-in by performing the *PIGA* correction assay using the HCT116-mut*PIGA* clone and modified sgRNAs. This assay, however, revealed that the incidence of targeted knock-in via TPN was in fact downregulated by both the truncation of spacers and the addition of two deoxyguanosines at the 5′ ends of spacers (Fig. 6a,b).

A recent study by Kim et al.^24^ showed that the cleaving capacity of Cas9 nuclease is affected by a single mismatched deoxyguanosine appended to the 5′ end of spacers to initiate transcription from a U6 promoter, and that the use of spacers that are exactly 20 nt in length (referred to as G/gN_19_, in which G and g represent a matched and mismatched deoxyguanosine at the 5′ end of spacers and N_19_ indicates that 19 matched nucleotides follow the 5′ terminal G/g) significantly enhances the cleaving activity of Cas9 nucleases compared with the use of 21-nt-long spacers (referred to as G/gN_20_). We therefore compared the capacity of G/gN_19_ and G/gN_20_ sgRNAs incorporated with Cas9 nickases to promote TPN-based targeted knock-in employing the *PIGA* correction assay as a platform. We found no significant difference in the efficiencies of TPN-based targeted knock-in achieved using modified sgRNAs #1 and #4 in the *PIGA* correction assay; yet, the average efficiency of targeted knock-in achieved using two G/gN_19_ sgRNAs is higher than, and that using two G/gN_20_ sgRNAs is lower than, that achieved using one G/gN_19_ and one G/gN_20_ sgRNAs. In addition, when sgRNA #2 was modified to a G/gN_19_ version and used together with sgRNA #3 in the *PIGA* correction assay, the efficiency of TPN-based targeted knock-in was slightly but significantly increased (Fig. 6c,d). These data collectively indicate that the use of G/gN_19_ sgRNAs marginally enhance the efficiency of TPN-based targeted knock-in and, among the distinct types of spacers assessed in this study, spacers exactly 20 nt in length promote the most efficient targeted knock-in via TPN.

## Discussion

In the present study, we showed that efficient targeted knock-in via TPN is supported by the use of 1700–2000-bp donor DNAs, sgRNAs predicted to be efficient in on-target cleavage, and tandem-paired Cas9 nickases with target sites located close together. In addition, sgRNAs should have exactly 20-nt-long spacers, and it seems beneficial to transfect cells with higher concentrations of donor DNAs along with relatively lower concentrations of Cas9 nickase-expressing plasmids for TPN. Overall, these findings will immediately help researchers to achieve higher efficiencies of targeted knock-in via TPN.

With regard to DNA strands being nicked, sense-strand nicking appeared to provide better efficiency of TPN-based targeted knock-in than antisense-strand nicking in *PIGA* correction assays (Figs. 1 and 6) and an EGFP-based assay (Fig. 2), but not in other EGFP-based assays (Fig. 3 and Supplementary Fig. S4). The reason for this difference between assays is unclear. However, because the distances between two nicking sites are larger in the *PIGA* correction assays (274 bp for #1–#4 and 242 bp for #2–#3) and the initial EGFP-based assay (60–704 bp; Fig. 2) than the latter EGFP-based assays (24–116 bp; Fig. 3 and Supplementary Fig. S4), a possible explanation may be that the strandedness (sense or antisense) of the nicked strand affects the efficiency of TPN-based targeted knock-in only when there is a substantial distance between the two nicks.

We previously described the similarity between the configuration of nicks created in TPN and the initial nicking step of Holliday’s theoretical model which explains meiotic DNA recombination in fungi^5,25,26^. Although the exact processes of DNA recombination elicited by TPN have yet to be elucidated, the experimental results from the present study offer further insights into DNA recombination triggered by TPN. For instance, the truncation of spacers from 20 nt to 17 or 18 nt and the addition of two deoxyguanosines at the 5′ ends of spacers often stimulate Cas9 nuclease-based targeted knock-in^22,23^ but attenuate TPN-based targeted knock-in, which provides evidence that distinct DNA recombination processes are promoted when different methods for targeted knock-in are used. In addition, we found that nicks placed close to the knock-in site support efficient TPN-based targeted knock-in, which suggests that gene conversion may be one mechanism that plays a major role in TPN-triggered DNA recombination.

As reported previously, TPN allows precise targeted knock-in without creating appreciable indels at on-target genomic sites or inducing p53-mediated DNA damage responses^5^. In addition, the use of Cas9 nickases for genome editing is known to drastically reduce indel formation at off-target genomic sites compared with the use of Cas9 nucleases^18,27,28^. Furthermore, targeted knock-in via TPN is accomplished with a relatively simple procedure using molecular tools that are common in genome editing (*i.e.*, Cas9 nickases and a donor DNA) and without the need to utilize extra biological materials. Accordingly, TPN-based targeted knock-in could potentially be used in the future in a wide range of applied life sciences, including clinical medicine and agriculture, and improving the efficiency of this technology will accelerate its practical application. However, despite the present study defining efficient TPN procedures, various experimental factors that will likely affect the efficiency of TPN-based targeted knock-in remain to be addressed. For instance, the overall efficiency of TPN-based targeted knock-in varies depending on recipient cell lines, yet the specific biological properties of cells that primarily determine the amenability of cells to TPN-based targeted knock-in are currently unknown. It is also unclear whether the classification of cells (*e.g.*, germline versus somatic, stem versus differentiated, and cancerous versus noncancerous) affects the efficiency of TPN-based targeted knock-in. Efficiency may also be affected by gene locus-dependent factors including the structure and chromosomal conformation of the genomic region harboring the target gene. As such, further studies are warranted to fully understand how the efficiency of TPN-based targeted knock-in is governed. In time, such studies will establish optimized TPN procedures and thereby enable highly efficient targeted knock-in.

## Methods

### General molecular biology techniques

Polymerase chain reaction (PCR) was performed with a Veriti thermal cycler (Thermo Fisher Scientific, Waltham, MA, USA) using KAPA HiFi HotStart ReadyMix (Roche, Basel, Switzerland). Plasmid DNA was isolated using the Mini Plus Plasmid DNA Extraction System (Viogene, New Taipei, Taiwan) according to the manufacturer’s protocol. For the isolation of transfection-grade plasmid DNA, the PureLink™ HiPure Plasmid Midiprep Kit (Thermo Fisher Scientific) was used. DNA sequencing was conducted using the BigDye Terminator v3.1 Cycle Sequencing Kit (Thermo Fisher Scientific) and electrophoresis was carried out on the 3500 genetic analyzer (Thermo Fisher Scientific).

### Plasmids

The expression plasmids SpCas9(BB)-2A-GFP (PX458) (#48,138), SpCas9(BB)-2A-Puro (PX459) V2.0 (#62,988), pSpCas9n(BB)-2A-GFP (PX461) (#48,140), and SpCas9n(BB)-2A-Puro (PX462) V2.0 (#62,987) were generous gifts from Dr. Feng Zhang (Broad Institute)^29^ provided via Addgene. To construct plasmids expressing a Cas9 nuclease and nickases, oligonucleotide pairs were designed, annealed to each other, and ligated to the BbsI-BbsI sites of PX459, PX461, or PX462 as per the Feng Zhang laboratory protocol. Protospacer sequences for the sgRNAs used in this study are listed in Supplementary Table S1.

Donor plasmids carrying *PIGA* homologous regions of six different lengths (Donor-*PIGA* plasmids) were generated by PCR amplification of homologous regions and the subsequent cloning of PCR products into pBluescript II KS ( +), except for Donor-*PIGA* (1991 bp) and Donor-*PIGA* (603 bp) that are identical to “Donor-*PIGA*” and “Donor-*PIGA*^short^” described in our previous study, respectively^5^. PCR-amplified regions within the newly constructed plasmids were verified by Sanger sequencing. The PCR primers used to generate donor plasmids in this study are listed in Supplementary Table S2. The donor plasmid used for *FLT3* gene editing, namely pcDNA/*FLT3*-ITD, was created in our previous study^17^.

prNCO, a plasmid stably transfected into the HCT116 and MCF10A cell lines to establish reporter cell clones that quantify the efficiency of targeted knock-in (namely, the HCT116-rNCO and MCF10A-rNCO clones), is identical to the pBABE-HR plasmid constructed and described in our previous studies^5,30^.

### Cell culture

The reporter cell clones used in this study (namely the HCT116-mut*PIGA*, HCT116-rNCO, and MCF10A-rNCO clones) were established and described in an earlier study^5^. HCT116-mut*PIGA* and HCT116-rNCO were maintained in McCoy’s 5A (modified) medium (Thermo Fisher Scientific) supplemented with 5% fetal bovine serum (FBS; Merck, Darmstadt, Germany) and 1% penicillin–streptomycin (P&S; Fujifilm, Tokyo, Japan). MCF10A-rNCO was cultured in D-MEM/Ham’s F-12 (Fujifilm) supplemented with 5% horse serum (Biowest, Nuaille, France), 20 ng/mL epidermal growth factor (Merck), 10 μg/mL insulin (Fujifilm), 500 ng/mL hydrocortisone (Merck), 100 ng/mL cholera toxin (Merck), and 1% P&S. 293T cells were cultured in Dulbecco’s modified Eagle’s medium (Fujifilm) supplemented with 10% FBS and 1% P&S. K562 cells and NALM-6 cells were cultured in Roswell Park Memorial Institute 1640 medium (Fujifilm) supplemented with 10% FBS and 1% P&S. Cell culture was carried out in a humidified incubator (PHCbi, Tokyo, Japan) maintained at 37 °C with 5% CO_2_.

For transfection of the derivative HCT116 clones and the K562 and NALM-6 cell lines, cells (1 × 10^6^ cells) were electroporated with plasmids (1 μg of each, unless otherwise noted) using the 4D-Nucleofector System (Lonza, Basel, Switzerland) according to the manufacturer’s instructions. The derivative MCF10A clone was plated onto 6-well tissue culture plates at a density of 2 × 10^5^ cells/well, and transfected with plasmids (1 μg of each) using Lipofectamine 3000 (Thermo Fisher Scientific) on the following day. PEI MAX (Polysciences, Warrington, PA, USA) was used to transfect 293T cells as per the manufacturer’s instruction.

### *PIGA* correction assay

The *PIGA* correction assay (correction of a *PIGA*-inactivating mutation) was carried out using the HCT116-mut*PIGA* clone, as previously described^5^. Briefly, the HCT116-mut*PIGA* clone was electroporated with PX459- or PX462-based constructs along with one of the Donor-*PIGA* plasmids carrying homologous regions of various lengths. The transfected clone was then incubated for 3 days before being stained with 0.1 µg/mL FLAER (Cedarlane, Ontario, Canada) in PBS containing 0.25 mM EDTA and 0.4% FBS. Subsequently, the cells were analyzed by FCM to determine the percentages of FLAER-positive cells using a FACSCanto II system (BD Biosciences, Franklin Lakes, NJ, USA). In parallel, in each experiment, the HCT116-mut*PIGA* clone was transfected with a GFP-expressing plasmid (PX458) along with Donor-*PIGA* (1991 bp) and then analyzed by FCM to determine “transfection efficiency.” The percentage of FLAER-positive cells in the other samples was divided by the determined transfection efficiency to calculate “FLAER-positive ratios” in the respective samples.

### rNCO assay

The rNCO assay (FCM-based detection of intramolecular EGFP-gene recombination within a construct) was carried out using the HCT116-rNCO and MCF10A-rNCO clones, as previously described^5^. Briefly, these clones were transfected with PX462-based constructs, allowed to grow for 3 days, and FCM-analyzed using a FACSCanto II system to determine the percentages of GFP-positive cells. In parallel, the reporter clones were transfected with the PX458 plasmid to determine transfection efficiency in each experiment and calculate the “GFP-positive ratios” in the other samples, similar to the procedure in the *PIGA* correction assay.

### IDAA assay

IDAA assays were performed as previously described^5,31–33^. In brief, IDAA assays evaluating the cleaving activity of Cas9 nucleases targeted to the *PIGA* gene (Supplementary Fig. S2) were initiated by transfecting 293T cells with PX459-based expression vectors and extracting gDNA from the transfected cells after three days of incubation. Nested PCR was then carried out to amplify and FAM-label a genomic region containing the Cas9 target sites using the primers listed in Supplementary Table S3. The resulting PCR products were electrophoresed on a 3500 Genetic Analyzer together with GeneScan 500 LIZ Size Standard (Thermo Fisher Scientific). Data were analyzed by GeneMapper software version 5.0 (Thermo Fisher Scientific) to determine the percentages of DNA fragments exhibiting altered amplicon size.

IDAA assays quantifying the targeted incorporation events of a 24-bp-long FLT3-ITD (Fig. 4) were carried out by transfecting K562 and NALM-6 cells with PX461-based vectors expressing Cas9 nickases together with the pcDNA/*FLT3*-ITD donor plasmid, and processing the transfected cells for FCM-based sorting of GFP-positive cells followed by a nested PCR amplification of the edited genomic region. The FAM-labeled PCR products were analyzed, and the percentages of DNA fragments bearing a 24-bp-long duplication were determined, as in the analyses of *PIGA* gene editing described above.

### Statistical analysis

All statistical analyses were performed using Intercooled Stata (StataCorp, College Station, TX, USA). One-way analysis of variance (ANOVA) with post hoc Bonferroni tests were performed to assess association between the lengths of homologous regions and the efficiency of TPN-based targeted knock-in (Fig. 1), association between the concentrations of plasmids used and the efficiency of TPN-based targeted knock-in (Fig. 5), and association between the use of the gN_19_ versions of *PIGA* sgRNAs #1 and #4 and the efficiency of TPN-based targeted knock-in (Fig. 6d). Differences in DNA cleaving activity among Cas9 nucleases designed against *PIGA* (Supplementary Fig. S2) were also assessed using one-way ANOVA. Wilcoxon rank-sum tests were applied to assess the significance of multiple experimental factors in the rNCO assays performed by nicking two closely located positions (Fig. 3 and Supplementary Fig. S4). A two-tailed unpaired t-test was performed to compare the gN_19_ and gN_20_ versions of *PIGA* sgRNA #2 for their capacity to facilitate TPN-based targeted knock-in (Fig. 6d). In all analyses, *p*-values of < 0.05 were considered statistically significant.

## Supplementary Information

Supplementary Information.

## Data Availability

Data and materials will be made available from the corresponding author upon reasonable request.
