# Supplementary material for: Experimental strategies to achieve efficient targeted knock-in via tandem paired nicking
Source: Sci Rep. 2021 Nov 19;11:22627. doi: 10.1038/s41598-021-01978-w (PMC8604973; doi:10.1038/s41598-021-01978-w)
Supplement: Supplementary file 1 — Supplementary Information. [file 41598_2021_1978_MOESM1_ESM.pdf]

Supplementary Information

for

Experimental strategies to achieve efficient targeted knock-in via tandem paired nicking

Md. Lutfur Rahman, Toshinori Hyodo, Sivasundaram Karnan, Akinobu Ota, Muhammad Nazmul Hasan, Yuko Mihara, Md Wahiduzzaman[§#], Shinobu Tsuzuki, Yoshitaka Hosokawa, and Hiroyuki Konishi*

Department of Biochemistry, Aichi Medical University School of Medicine, Nagakute, Aichi 480-1195, Japan

[§] Current address: *Bangladesh Medical Research Council, Dhaka-1212, Bangladesh*

[#] Current address: *Eukaryotic Gene Expression & Function (EuGEF) Research Group, Chattogram-4000, Bangladesh*

* Corresponding author
Hiroyuki Konishi
Department of Biochemistry, Aichi Medical University School of Medicine
1-1 Yazako-Karimata, Building #2, Room 362,
Nagakute, Aichi 480-1195, Japan
Phone: +81-561-62-3311, ext. 12362
Fax: +81-561-61-4056
E-mail: hkonishi@aichi-med-u.ac.jp

CONTENTS

- **Supplementary Figures S1–S4**
- **Supplementary Tables S1–S3**

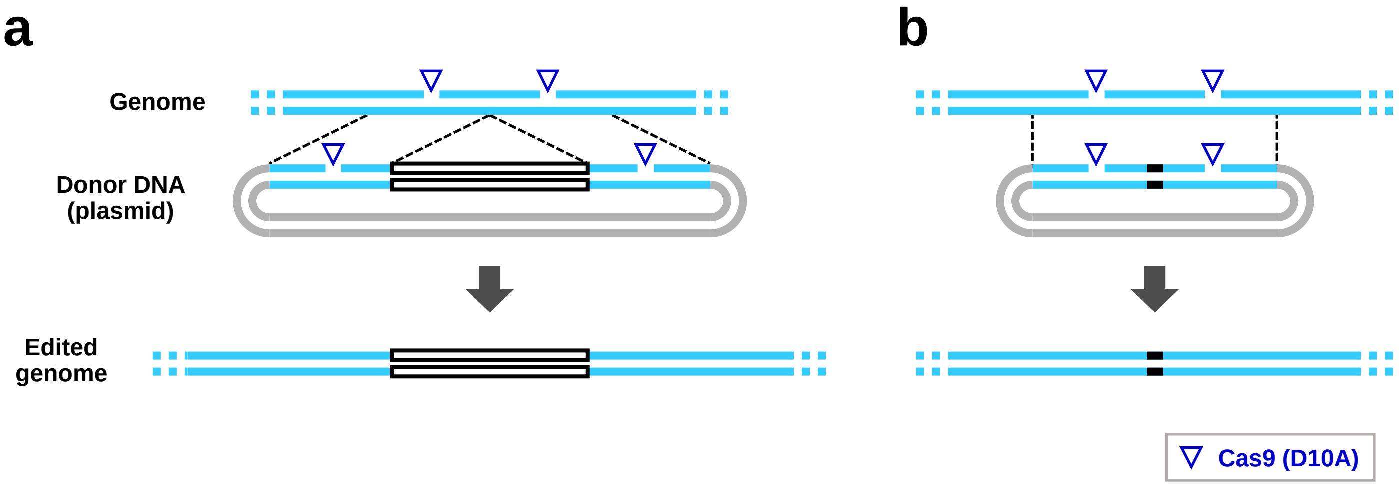

Supplementary Figure S1

Schematic representation of TPN-based targeted knock-in.

(a) Targeted insertion of a large DNA fragment (open boxes) by TPN.

(b) Introduction of a nucleotide substitution (black dots) by TPN. Blue lines: genomic sequences; gray lines: plasmid backbones.

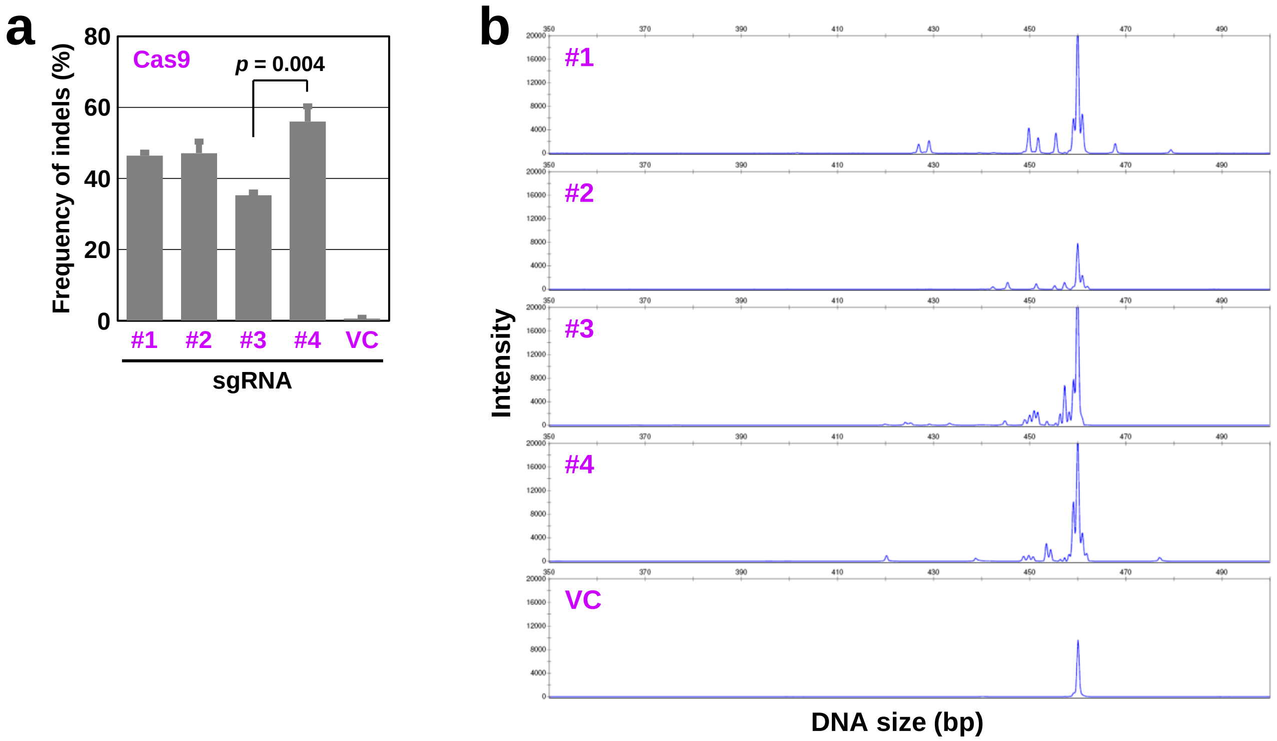

Supplementary Figure S2
Cleaving capacity of Cas9 nucleases designed against the *PIGA* gene. Cas9 nucleases #1–#4, which contain the same sgRNAs as Cas9 nickases #1–#4 (Fig. 1a) respectively, were transfected into 293T cells. IDAA assays were then performed to evaluate the cleaving capacity of the Cas9 nucleases.

(a) Graphical representation of all results. Data represent the mean and SEM values of three independent experiments.

(b) Representative chromatographs. PCR amplicons without size alteration are 461 bp in size.

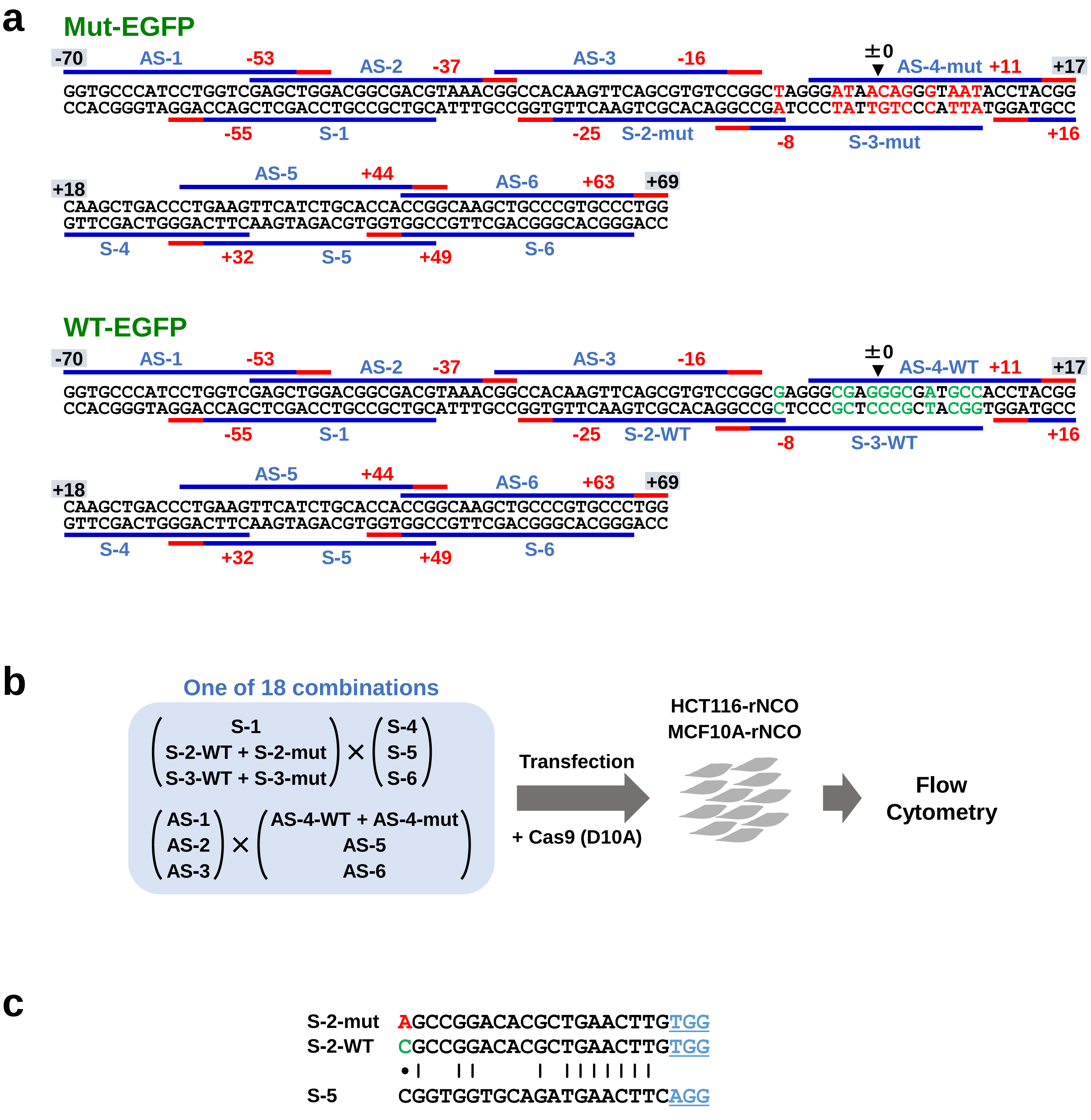

Supplementary Figure S3

Experimental design for the evaluation of sgRNAs targeted close to the knock-in site on the EGFP gene.

(a) DNA sequences of the mutant and wild-type EGFP gene within the 70-bp range from the knock-in site (±0). Blue and red bars indicate 20-bp protospacers and adjacent PAM sequences, respectively. Numbers in red indicate the positions of nicked sites. Nucleotides shown in red and green represent a mutated portion of EGFP and its corresponding wild-type sequence, respectively.

(b) Schematic representation of the assay.

(c) High similarity found in S-2 and S-5, a combination of 20-bp spacers used in this assay. Vertical bars indicate complete identity among three spacers, whereas the dot indicates identity only between two spacers (S-2-WT and S-5). Blue underlined letters represent PAM sequences.

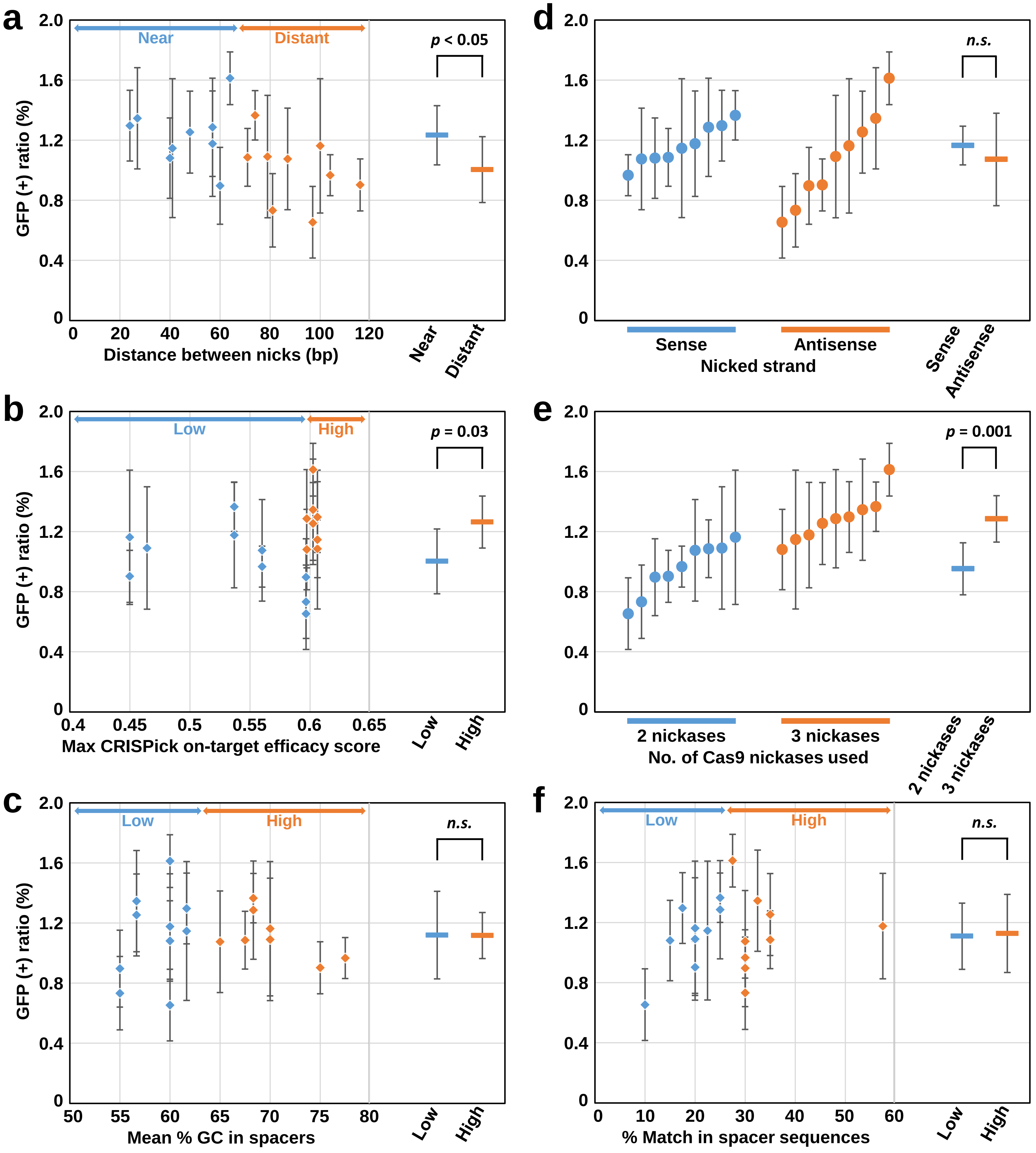

Supplementary Figure S4

Efficiencies of targeted knock-in via TPN assessed using MCF10A-rNCO and sgRNAs targeted close to the knock-in site in the EGFP gene. Data are graphically represented in a similar manner to the graphs shown in Figure 3. Data represent mean and standard deviation of three independent experiments (left in each panel) or 18 sgRNA combinations divided into two groups (right in each panel). For experimental details, see Supplementary Fig. S3a, b.

Supplementary Table S1: Protospacers and PAM sequences employed in this study

Protospacer name	Sequence
PIGA-nickase-1	5′-AAACAATGACCCGATCCTGCAGG
PIGA-nickase-2	5′-TTTATCATGGGACAGGTGATGGG
PIGA-nickase-3	5′-GATGTTGCAATAGATGCCACTGG
PIGA-nickase-4	5′-TTATTAGTCCAGGCACCCCGTGG
PIGA-nuclease-A	5′-GGTATATGACCGGGTATCAGTGG
EGFP-nickase-1	5′-ATCGCCAAAAAAGAAGAGAAAGG
EGFP-nickase-2	5′-CGTCGCCGTCCAGCTCGACCAGG
EGFP-nickase-3	5′-AAGAGGGCCAAGCACCCCCCCGG
EGFP-nickase-4	5′-CTCCTGGGCTTCTCGGTGCCGGG
EGFP-nickase-5	5′-CGGTGGATCCACGCCGGGCTTGG
EGFP-nickase-6	5′-GCACTTCGCCAAGCCCGGCGTGG
EGFP-nickase-7	5′-CGTCGCCGTCCAGCTCGACCAGG
EGFP-nickase-8	5′-GGCCACAAGTTCAGCGTGTCCGG
EGFP-nickase-9	5′-GGCCACAAGTTCAGCGTGTCCGG
EGFP-nickase-10	5′-CTGAAGTTCATCTGCACCACCGG
EGFP-nickase-11	5′-CGTGCTGCTTCATGTGGTCGGGG
EGFP-nickase-12	5′-TTCAAGTCCGCCATGCCCGAAGG
EGFP-nickase-13	5′-CAACTACAAGACCCGCGCCGAGG
EGFP-nickase-14	5′-TCAGCTCGATGCGGTTCACCAGG
EGFP-nickase-15	5′-CAACATCCTGGGGCACAAGCTGG
EGFP-nickase-16	5′-CGGTGGTGCAGATGAACTTCAGG
FLT3-nickase-1	5′-CACTATTTATAATGTCACACAGG
FLT3-nickase-2	5′-GTACAAAAAGGTAAAAGCAAAGG
FLT3-nickase-3	5′-GTATGAAAGCCAGCTACAGATGG
FLT3-nickase-4	5′-ACTCACCATTTGTCTTTGCAGGG
FLT3-nickase-5	5′-GTTGCCGTCAAAATGCTGAAAGG
FLT3-nickase-6	5′-CAAAGATGCACAAAAATGGGAGG

PAM: protospacer adjacent motif

Underlining indicates PAM sequences.

Additional EGFP protospacers depicted in Supplementary Fig. S3 are not listed in this table.

Both Cas9 nickases and nucleases were created against *PIGA*-nickase-1–4.

Supplementary Table S2: PCR primers used to amplify homologous regions of various lengths within the *PIGA* gene in preparation for the creation of donor plasmids

Primer name	Sequence
*PIGA*603-F*	5′-GCTGTCTAGACTTTGCGGGACTTTGGA
*PIGA*603-R*	5′-TCTACTCGAGCTTCTACCTGGTTTCAGATA
*PIGA*895-F	5′-AGATGCTCTAGAGAGCATCTCAGCTTAGTC
*PIGA*895-R	5′-CAAGCTCGAGCTACACTCAGGAATTGCATAC
*PIGA*1161-F	5′-CCTCACTCTAGATGCTCAGCAACATGTACAG
*PIGA*1161-R	5′-TGGCCTCGAGATGCCCAGAAAACGTTTGGC
*PIGA*1442-F	5′-GCTCAGTCTAGAACGTTATTTCATTTGCCAGC
*PIGA*1442-R	5′-TTACCTCGAGCACCTACTGAGTGAACCTAAT
*PIGA*1722-F	5′-TACAAATCTAGAATGTTGGCCCATTTGATGT
*PIGA*1722-R	5′-CACACTCGAGATGAAGTAAGCCATTTGAATGG
*PIGA*1991-F*	5′-GTACCTTCTAGAGGCTGTTGACCTGTACAACA
*PIGA*1991-R*	5′-AAAAAACTCGAGCACAAACACAACTGAAGATGG

PCR: polymerase chain reaction

Underlining indicates the restriction enzyme sites used for the incorporation of PCR products into a plasmid.

Asterisks indicate the primers used to create donor plasmids in our previous study[5].

Supplementary Table S3: PCR primers used for IDAA assays

Primer name	Sequence
PIGA-IDAA-1st-F	5′-GCTCGATGAAAACATGCCGTACCT
PIGA-IDAA-1st-R	5′-ACAGTGATATCGGTCCCCAG
PIGA-IDAA-2nd-F	5′-AGCTGACCGGCAGCAAAATTGGGATATCATTACTATGACAAC
PIGA-IDAA-2nd-R	5′-TTACAATCTAGGCTTCCTTCTAC
FLT3-IDAA-1st-F	5′-AGCCTCCTTATTTGCCCTCAG
FLT3-IDAA-1st-R	5′-AAGAGAAGAAGGCATGGGTGG
FLT3-IDAA-2nd-F	5′-AGCTGACCGGCAGCAAAATTGCCAGCTACAGATGGTACAGGT
FLT3-IDAA-2nd-R	5′-TGGGAAACTGTGCCTCCCAT
FamFwd	5′-(6-FAM)-AGCTGACCGGCAGCAAAATTG

IDAA: indel detection by amplicon analysis
